# Alkaloids Analysis of *Habranthus cardenasianus* (Amaryllidaceae), Anti-Cholinesterase Activity and Biomass Production by Propagation Strategies

**DOI:** 10.3390/molecules26010192

**Published:** 2021-01-02

**Authors:** Daniel Zaragoza-Puchol, Javier E. Ortiz, Alejandro A. Orden, Marianela Sanchez, Jorge Palermo, Alejandro Tapia, Jaume Bastida, Gabriela E. Feresin

**Affiliations:** 1 Instituto de Biotecnología, Universidad Nacional de San Juan, Av. Libertador General San Martín 1109 (O), C.P. 5400 San Juan, Argentina; josedanielzaragoza@gmail.com (D.Z.-P.); jortiz@unsj.edu.ar (J.E.O.); atapia@unsj.edu.ar (A.T.); 2 Consejo Nacional de Investigaciones Científicas y Técnicas (CONICET), Godoy Cruz 2290, C.P. 1425 Ciudad Autónoma de Buenos Aires, Argentina; 3 INTEQUI CONICET, Facultad de Química Bioquímica y Farmacia, Universidad Nacional de San Luis, Almirante Brown 1455, C.P. D5700HHW San Luis, Argentina; alejandroorden@gmail.com; 4 Departamento de Química Orgánica, Facultad de Ciencias Exactas y Naturales, Universidad de Buenos Aires, Ciudad Universitaria, Pabellón, C.P. 1428 Buenos Aires, Argentina; sanchezmnela@gmail.com (M.S.); palermo@qo.fcen.uba.ar (J.P.); 5 CONICET–Universidad de Buenos Aires, Unidad de Microanálisis y Métodos Físicos en Química Orgánica (UMYMFOR), Ciudad Universitaria, C.P. 1428 Buenos Aires, Argentina; 6 Centro de Investigaciones y Transferencia de Villa María (CITVM-CONICET), Universidad Nacional de Villa María, Campus Universitario, Arturo Jauretche 1555, Villa María, C.P. 5000 Córdoba, Argentina; 7 Instituto de Ciencias Básicas, Universidad Nacional de San Juan, Av. Libertador General San Martín 1109 (O), C.P. 5400 San Juan, Argentina; 8 Departament de Biologia, Sanitat i Medi Ambient, Facultat de Farmàcia i Ciències de l’Alimentació, Universitat de Barcelona, Avda. Joan XXIII # 27–31, 08028 Barcelona, Spain; jaumebastida@ub.edu

**Keywords:** Amaryllidaceae, bioactive alkaloids, GC-MS, propagation methods, biomass production

## Abstract

Plants in the Amaryllidaceae family synthesize a diversity of bioactive alkaloids. Some of these plant species are not abundant and have a low natural multiplication rate. The aims of this work were the alkaloids analysis of a *Habranthus cardenasianus* bulbs extract, the evaluation of its inhibitory activity against cholinesterases, and to test several propagation strategies for biomass production. Eleven compounds were characterized by GC-MS in the alkaloid extract, which showed a relatively high proportion of tazettine. The known alkaloids tazettine, haemanthamine, and the epimer mixture haemanthidine/6-epi-haemanthidine were isolated and identified by spectroscopic methods. Inhibitory cholinesterases activity was not detected. Three forms of propagation were performed: bulb propagation from seed, cut-induced bulb division, and micropropagated bulbs. Finally, different imbibition and post-collection times were evaluated in seed germination assays. The best propagation method was cut-induced bulb division with longitudinal cuts into quarters (T1) while the best conditions for seed germination were 0-day of post-collection and two days of imbibition. The alkaloids analyses of the *H. cardenasianus* bulbs showed that they are a source of anti-tumoral alkaloids, especially pretazettine (tazettine) and T1 is a sustainable strategy for its propagation and domestication to produce bioactive alkaloids.

## 1. Introduction

A large number of modern medicinal drugs are obtained from plant extracts [1]. The use of native plants as a source of bioactive molecules motivates studies of their prospection, propagation, and domestication [2]. The Amaryllidaceae family is a large group of 1100 species divided among 75 genera, which are used for floricultural-ornamental and medicinal purposes [3]. Species belonging to Amaryllidaceae have also been used in traditional medicine by indigenous people throughout the world to cure ailments: stomach ailments, skin diseases, headaches, dizziness, wounds, chest and bladder pain, renal and liver complaints, infertility, aching joints, rheumatism, snake bites, facilitation of childbirth during labor, hysteria, and as narcotics [4]. In Argentina, the bulbs and leaves of *Hippeastrum parodii* Hunz. & Cocucci and *Zephyranthes carinata* Herb. (Amaryllidaceae) are used in traditional medicine by the Toba indigenous community for the treatment of skin disorders (warts, facial pimples, acne, and skin spots) [5]. The alkaloid galanthamine is a long-acting, selective, reversible, and competitive acetylcholinesterase (AChE) inhibitor used for the treatment of Alzheimers disease and is still obtained from plants, mainly from *Leucojum aestivum* (summer snowflake) and Narcissus (daffodils) in Europe, *Lycoris radiata* in China, and *Ungernia victoris* in Tadzhikistan and Uzbekistan [6,7]. Amaryllidaceae family belongs to the monocot order Asparagales and is composed of three subfamilies: Amaryllidoideae, Agapanthoideae, and Allioideae. In particular, the Amaryllidoideae subfamily is an important source of bioactive molecules [8].

*Habranthus* Herbert is a widely distributed Amaryllidaceae genus in America, consisting of 25 to 30 species [9]. These species biosynthesize alkaloids from the precursor 4′-*O*-methylnorbelladine, which is derived from the amino acids L-phenylalanine and L-tyrosine [10]. The nomenclature of the Amaryllidaceae skeleton types, also referred to as structural types, subgroups, ring types or series, has varied over the years [3]. In this work, the Amaryllidaceae alkaloids (AA) are classified into seventeen skeleton types according to Berkov et al. [3].

A display several types of pharmacological activities, most importantly on the central nervous system, including neuroprotection against the excitotoxicity induced by glutamate in primary cortical neurons, antiproliferation, cytotoxicity, and apoptosis induction [11,12]. Anti-viral, anti-parasitic, anti-cancer, and anti-cholinesterasic activities have also been reported [3,11,13,14,15].

Amaryllidaceae species, including *Habranthus cardenasianus* Traub & I.S. Nelson, are not abundant, and present a low natural multiplication rate [16]. Mining and agricultural activities, together with urban growth, are reducing the populations of plants in this family. Thus, the extraction of bioactive metabolites from natural sources is currently not viable, and it is important to develop sustainable propagation methods for Amaryllidaceae domestication [17]. Such methods include seed propagation [18,19], cut-induced bulb division [20,21,22], and micropropagation [23,24,25]. Seed propagation is a simple and economical way to obtain seedlings [26] and allows a good multiplication rate, but it has the drawback of a high genetic drift [19]. Cut-induced bulb division consists of making incisions that induce hormonal mechanisms in the plant and promote the generation of new bulbils [20]. In-vitro culture is large scale micropropagation using tissue culture techniques [23]. Micropropagation is possible due to the totipotentiality of plant cells, which is the ability to regenerate a complete plant under the appropriate stimuli [17,24]. Micropropagation is very useful in breeding programs because it produces a uniform quality of commercial plants, with a high multiplication rate, little genetic variability, and a stable production of the metabolites of interest [23]. To the best of our knowledge, this work represents the first chemical study of *H. cardenasianus* bulbs, the evaluation of their inhibitory activity against cholinesterases, and the analysis of biomass production by several propagation strategies to produce bioactive alkaloids.

## 2. Results and Discussion

### 2.1. Chemical Analysis

#### 2.1.1. Alkaloid Profile by GC-MS Analysis

The alkaloid extract analysis showed eleven compounds, nine of them identified by comparison of their mass fragmentation patterns with EI-MS spectra home-made database gathered from alkaloids previously isolated and identified by different spectroscopic methods (1D and 2D NMR, UV, CD, IR, HRMS) in the Natural Products Laboratory of Barcelona University. Additionally, Kovats retention index (RI) values, the NIST 05 Database, and literature data were used for alkaloid identification. Besides the known AA, two compounds could not be identified. The GC-MS data are shown in Table 1. Based on the AA classification of Berkov et al. [3] the identified compounds in *H. cardenasianus* are galanthindole (galanthindole), haemanthamine (haemanthamine, haemanthidine/6-epi-haemanthidine), homolycorine (two *m*/*z* 109 unknown alkaloids), ismine (ismine), lycorine (anhydrolycorine, 11,12-dehydroanhydrolycorine, lycorine), narciclasine (5,6-dihydrobicolorine), and pretazettine (tazettine) type alkaloids [3]. The unknown alkaloids were tentatively identified as homolycorine-type due to their fragmentation pattern, which showed a characteristic *m*/*z* 109 (100) base peak. The alkaloid tazettine represented more than 50% of the total ion current (TIC). It is known that tazettine is not a naturally occurring alkaloid, but an artifact produced from the chemically labile parent alkaloid pretazettine arising from a rearrangement during alkaloid extraction [27,28,29]. Thus, considering the basicity and reflux conditions during the *H. cardenasianus* extract preparation, it would be very likely that pretazettine was totally converted to tazettine.

Based on the TIC proportions, the most abundant alkaloid skeletons were the pretazettine, haemanthamine, and homolycorine types. The relative proportion of each alkaloid was determined as a percentage of the TIC. Thus, the major components were identified as tazettine (9) (57.77%), haemanthamine (8) (7.92%), and haemanthidine/6-epi-haemanthidine (10) (6.68%) (Table 1).

The main groups of hemanthamine, pretazettine, and narciclasine alkaloids originate from a para-para’ oxidative phenolic coupling of 4′-*O*-methylnorbelladine [27]. Thus, taking into account the relative TIC proportions of these alkaloid types, it is likely that the metabolism of *H. cardenasianus* favors alkaloid biosynthesis via this oxidative pathway. On the other hand, galanthamine-type alkaloids were not detected.

**Figure 1:**
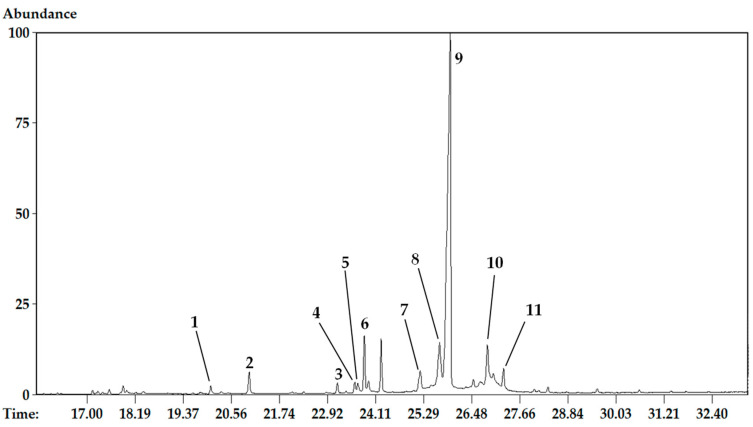
GC chromatogram of *H. cardenasianus* extract showing the alkaloids identified by MS. (**1**) Ismine, (**2**) 5,6-Dihydrobicolorine, (**3**) Unknown, (**4**) Galanthindole, (**5**) Anhydrolycorine, (**6**) Unknown, (**7**) 11,12-dehydro anhydrolycorine, (**8**) Haemanthamine, (**9**) Tazettine, (**10**) Haemanthidine/6-epi-haemanthidine, (**11**) Lycorine.

Alkaloids are a family of compounds of great interest in cancer treatment due to their anti-proliferative and anti-angiogenic effects. The structural diversity of alkaloids and their capacity to interact with a wide spectrum of molecules is due to the different amino acids that act as precursors for their biosynthetic pathways. [30]. AA are particularly diverse, and share norbelladine as a common biosynthetic precursor, which is obtained from the condensation of the aromatic amino acids L-phenylalanine and L-tyrosine. These alkaloids have undoubtedly revolutionized medicine as inhibitors of cholinesterases, and as antiviral, anti-inflammatory, antibacterial, and most importantly, antitumor agents, serving as a reservoir of valuable anticancer drugs [31]. The pharmaceutical potential of AA has been confirmed by the registration of galanthamine, an inhibitor of AChE, as a drug for Alzheimer’s disease [6,32]. Other AA are of commercial interest, but are available only in low amounts in the native plants. The most abundant alkaloids isolated from *H. cardenasianus* were tazettine (9) (pretazzetine), haemanthamine (**8**), and haemanthidine/6-epi-haemanthidine (10) (Figure 1), all of which have been reported as bioactive compounds [3,11].

A major reason for the failure of cancer treatment is multidrug resistance (MDR), which in most cases is caused by the activity of the various ABC transporters and p-glycoproteins encoded by MDR genes, which pump anticancer drugs out of the cells [33]. The efficiency of cytotoxic agents that target cancer cells can be enhanced by the inhibition of P-gp, one of the most important ABC proteins. Some AA exhibit a combination of anticancer activities, including a direct cytostatic effect and P-gp-inhibition, and have been proposed as promising starting structures for the development of future anticancer agents [34]. Pretazettine acts on several cancer cells by affecting the p-glycoprotein ABCB1, an interesting and uncommon molecular mechanism [27,33]. In addition, it displays excellent antiproliferative effects on both human and mouse cell lines, indicating that pretazettine-type alkaloids are an attractive option for alkaloid-based anticancer therapy [34].

There is compelling evidence for the interaction of tazettine alkaloids with macromolecules such as DNA, RNA, and proteins, but their potential role in other areas of molecular cancer chemotherapeutics such as apoptosis still needs to be investigated. A recent review by Nair & Van Staden [35] informs that eleven of the AA from eight Amaryllidaceae genera have been assayed against as many as 50 different cancer cell lines. It has been shown that pretazettine is capable of inhibiting cellular protein synthesis without affecting the synthesis of DNA or RNA in KB human oral epidermoid carcinoma, P-388, and Ehrlich ascites cells. When tested against different tumoral cell lines (the HeLa cervical adenocarcinoma cell line, mouse melanoma cells, Molt4 T-lymphoma cells, L929, and MCF7 cells), pretazettine was found to be far more potent than tazettine (9). Furthermore, pretazettine activity against Rauscher leukemia has been reported in BALB/c mice [36].

Due to its high content of pretazettine, *H. cardenasianus* is a promising source of this compound. According to Nair & Van Staden [35], pretazettine is one of the most active AA against Molt4 lymphoid cells, with particularly striking activity at the submicromolar level, suggesting it may have application in lymphoma chemotherapeutics. Pretazettine also possesses pronounced activity against Herpes simplex type 1 virus, which could reflect a general ability to inhibit protein synthesis during viral replication [27]. Additionally, previous reports show that tazettine (9) and pretazettine are relatively safe in animal models [27,35].

Promising antiproliferative activities have been described for haemanthamine (8) and haemanthidine (10) [36], both identified in *H. cardenasianus* bulbs. Haemanthamine (8) displays significant in vitro cytotoxic activity against several types of cancer cell lines, including MoltT-4, HepG2, HeLa, MCF7, CEM, K562, A549, Caco-2, HT-29, A2780, SW1573, and T47-D, whereas haemanthidine (**10**) has been reported as a promising cytotoxic agent against A549, OE21, Hs683, and SKMEL cancer cells, and has a growth inhibitory effect on both parental and multidrug resistant L5178 mouse lymphoma cell lines [12]. Haemanthidine is also anti-inflammatory in mice and has higher analgesic activity than aspirin or indomethacin [37]. Cedrón et al. [38] suggested that some haemanthamine derivatives are effective inhibitors of the malaria-causing parasite *Plasmodium falciparum* and could provide a useful starting point for the preparation of new haemanthamine-type derivatives with improved antiplasmodial activity. Additionally, Nair & van Staden [39] report better overall activities for haemanthidine (10) against various strains of *P. falciparum*, particularly the K1 strain (IC_50_ 0.35 μg/mL). The alkaloids haemanthamine (8) and haemanthidine (10) were the most active compounds against *P. falciparum* and have served as useful scaffolds for structure-activity relationship studies, affording new insights into the antiplasmodial pharmacophore and the features supporting its efficacy.

#### 2.1.2. Alkaloid Isolation

**Figure 2:**
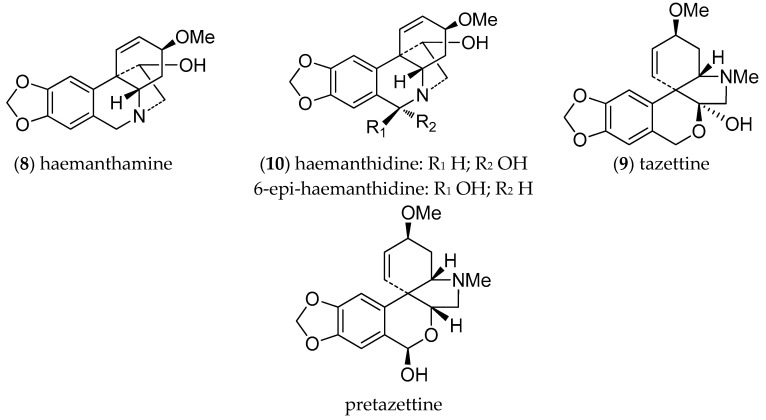
Alkaloids from *H. cardenasianus*.

Extensive chromatographic purification afforded the known alkaloids haemanthamine (8) (12 mg), tazettine (9) (89 mg), and the haemanthidine/6-epi-haemanthidine mixture (10) (8 mg) (Figure 2). Their structures were confirmed by NMR spectroscopy showing the presence of a mixture of both epimers for alkaloid 10. The NMR results are in agreement with literature data [27]. Likewise, for the alkaloid haemanthidine (10), previous reports indicate that in many Amaryllidaceae species this alkaloid occurs naturally as a mixture of C-6 epimers [3,27,38,40].

### 2.2. AChE and Butyrylcholinesterase (BuChE) Inhibitory Activities

The alkaloid extract from *H. cardenasianus* and the isolated alkaloids were evaluated in vitro for AChE and BuChE inhibitory activities. Galanthamine (Sigma-Aldrich) was used as a positive control, showing an IC_50_ value of 1 µM. The isolated alkaloids 8, 9, and the epimeric mixture 10 had IC_50_ values higher than 200 µM, and were therefore considered inactive, in agreement with previous data [41], as was the alkaloid extract, which had IC_50_ values of 250 µg/mL and 300 µg/mL for AChE and BuChE inhibition, respectively. Galanthamine-type alkaloids are recognised as the most potent cholinesterases inhibitors among the AA, and their non-detection by GC-MS analysis in the *H. cardenasianus* extract supports the results of the cholinesterase inhibition assays.

### 2.3. Propagation from Habranthus cardenasianus

Collected material of *H. cardenasianus* was used for vegetative and sexual propagation experiments to generate biomass. Five different propagation methods were applied: T0 (control, whole bulb without cuts), T1 (bulb quarters), T2 (twin scales), T3 (bulb basal disc); T4 (micropropagated bulbs), and T5 (seeds). Seven months later, the following parameters were evaluated: survival percentage, multiplication rate, biomass increase, bulb equatorial diameter and length; and root diameter, length, and number.

**Figure 3:**
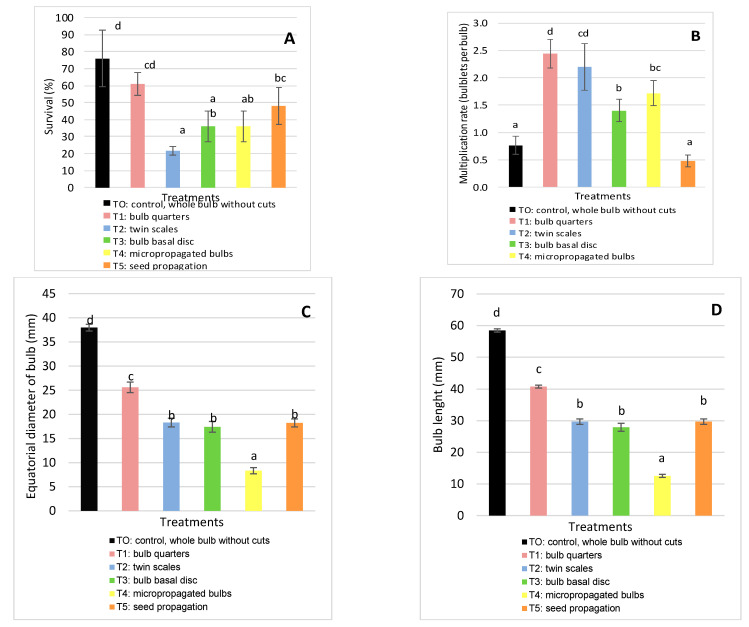
Effect of the propagation method on survival (**A**); multiplication rate (**B**); equatorial diameter of bulb (**C**); and bulb length (**D**). Means and standard deviation are reported. Data were analyzed by ANOVA and different letters (a, b, c, d) indicate significant differences by the Tukey test (*p* < 0.05). The treatments were: T0 (Control): whole bulb, T1: bulb quarters, T2: twin scales, T3: bulb basal discs, T4 micro propagated bulbs and T5: seed propagated bulbs.

The coefficients of variation were less than 10%, the determination coefficients were greater than 0.90 and p-values of the model were less than 0.05, which indicated little variation in the observations and a good fit of the statistical model ANOVA. The obtained data demonstrate that the propagation method can significantly affect all the evaluated parameters (*p* < 0.05). The maximum biomass was recorded in cultivated plants. The different propagation methods had a significant influence on survival: T0 (control, whole bulb without cuts) and T1 (bulb quarters) presented the highest percentage of surviving bulbils (76 ± 6.73 % and 61 ± 6.52%, respectively) and T3 (twin scales) the lowest (Figure 3A). Witomska et al. [22] reported that the reserves in the cataphylls of the mother bulb play an important role in the survival of new bulbils [16].

Another parameter significantly affected during the propagation assays was the multiplication rate, which was highest in T1 (bulb quarters) and T2 (twin scales) (2.44 ± 0.26 and 2.20 ± 0.42 bulbils per mother bulb, respectively).

The lowest multiplication rate was observed in T0 (control, whole bulb without cuts) and T5 (seed propagation) (0.76 ± 0.17 and 0.48 ± 0.11 bulbils per mother bulb, respectively) (Figure 3B). Andrade-Rodriguez et al. [16] reported similar findings, indicating that the presence of the main bud in the bulbs inhibited the development of new bulbils, whereas complete longitudinal cuts promoted bulbil production. Additionally, Witomska et al. [22] showed that cuts in the basal disc are essential to stimulate bulbil production. However, in experiments with *Hippeastrum × johnsonii* bulbs, Kharrazi et al. [20] observed that the largest number of bulblets was produced by the twin scaling method, although they took longer to grow to a final, commercial size.

The equatorial bulb diameter and bulb length differed significantly according to the propagation methods applied, being largest in T0 (control, whole bulb without cuts) and T1 (bulb quarters). The equatorial diameter values for T0 and T1 were 37.96 ± 1.69 mm and 25.62 ± 1.09 mm, respectively, whereas the bulb length values were 58.46 ± 1.53 mm and 40.76 ± 1.46 mm, respectively. No significant differences were observed between T2 (twin scales), T3 (bulb basal disc) and T5 (seed propagation), while T4 (micropropagated bulbs) produced the smallest values (Figure 3C,D).

According to Kharrazi et al. [20] and Letelier & Cabello [21], large bulb fractions have greater reserves and so generate larger bulbils. Also, Andrade-Rodriguez et al. [16] reported that the biggest bulbils were obtained after applying the four longitudinal section treatment, whereas the smallest bulbils were produced by the twin cataphyll method.

**Figure 4:**
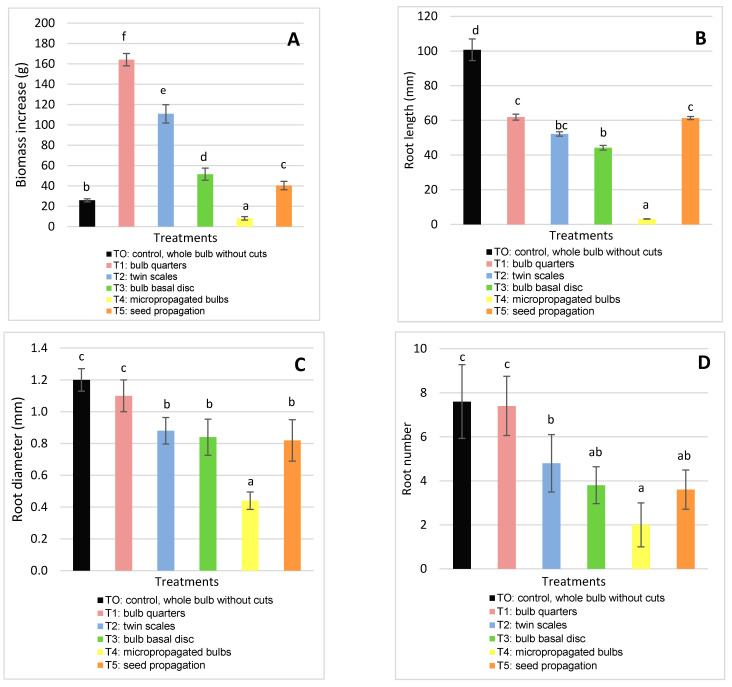
Effect of the propagation method on biomass increase (**A**); root diameter (**B**); root length (**C**); and root number (**D**). Means and standard deviation are reported. Data were analyzed by ANOVA and different letters (a, b, c, d) indicate significant differences by the Tukey test *(p* < 0.05). The treatments were: T0 (Control): whole bulb, T1: bulb quarters, T2: twin scales, T3: bulb basal discs, T4 micro propagated bulbs and T5: seed propagated bulbs.

Regarding biomass increase, significant differences were observed between all the treatments. The treatments that resulted in the highest survival and multiplication rate as well as the largest bulb diameters and lengths produced the most biomass. The highest biomass production was obtained in T1 (bulb quarters): 164.15 ± 6.04 g (Figure 4A), and the lowest in T0 (control, whole bulb without cuts) and T4 (micropropagated bulbs). The starch of the mother bulbs is the reserve substance used in the initial growth phase, until bulbs have photosynthetically active leaves [42].

**Figure 5:**
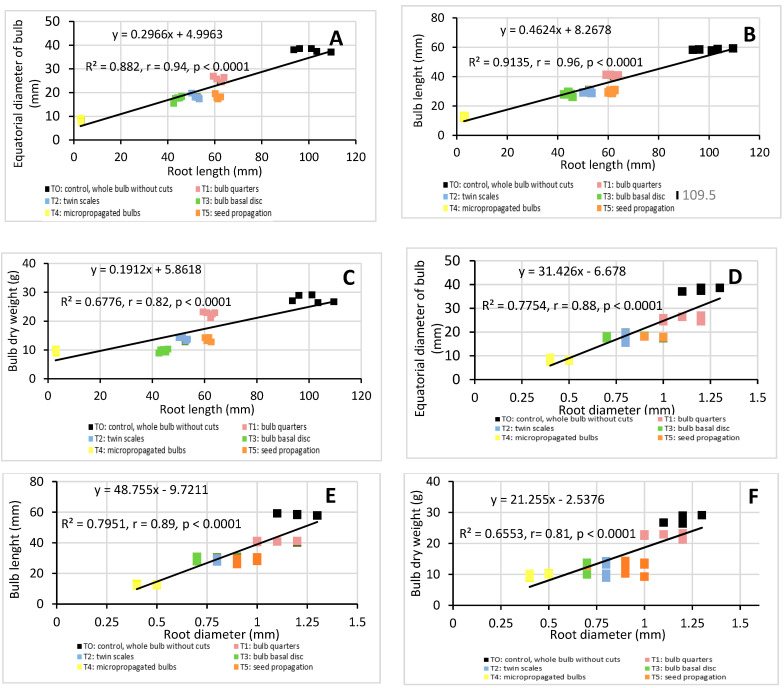

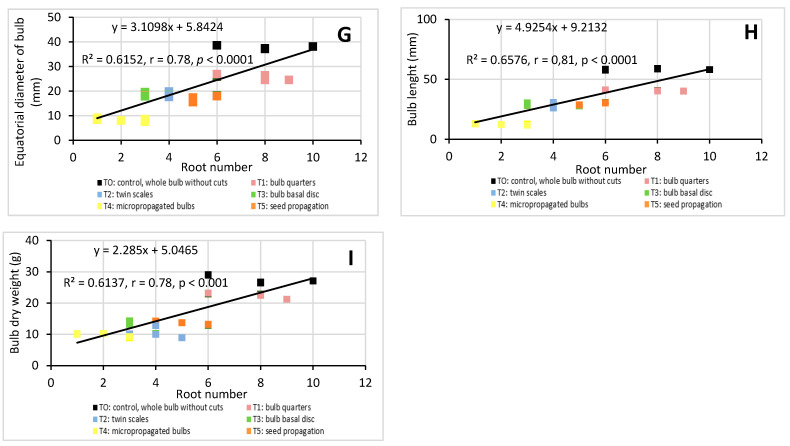
Linear correlation analysis: root length-equatorial bulb diameter (**A**), root length-bulb length (**B**), root length-bulb dry weight (**C**), root diameter-equatorial bulb diameter (**D**), root diameter-bulb length (**E**), root diameter-bulb dry weight (**F**), root number-equatorial bulb diameter (**G**), root number-bulb length (**H**), root number-bulb dry weight (**I**). Coefficient of determination (R^2^) and Pearson’s correlation coefficient (r) are reported. *p* < 0.05 indicate that the linear correlation is statistically significant.

Although root parameters (length, diameter, and number) are important factors in determining the best method to propagate bulb plants [20], they are rarely mentioned in previous studies. In order to demonstrate the importance of roots in *H. cardenasianus* propagation, a linear correlation analysis was applied (Figure 5). There was a significant correlation between root parameters and all the biomass parameters. The root length had a high positive linear correlation with the equatorial bulb diameter (R^2^ = 0.88, r = 0.94, *p* < 0.0001) and the bulb length (R^2^ = 0.91, r = 0.96, *p* < 0.0001) (Figure 5A,B). The root length-bulb dry weight correlation presented the following values: R^2^ = 0.68, r = 0.82, *p* < 0.0001 (Figure 5 C). The root diameter also presented a high positive linear correlation with the bulb equatorial diameter (R^2^ = 0.78, r = 0.88, *p* < 0.0001) and bulb length (R^2^ = 0.80, r = 0.89, *p* < 0.0001) (Figure 5D,E). The root diameter-bulb dry weight correlation presented the following values: R^2^ = 0.66, r = 0.81, *p* < 0.0001 (Figure 5F). The root number also showed a positive linear correlation with the equatorial bulb diameter (R^2^ = 0.62, r = 0.78, *p* < 0.0001), bulb length (R^2^ = 0.66, r = 0.81, *p* < 0.0001), bulb dry weight (R^2^ = 0.62, r = 0.78, *p* < 0.0001), and bulb length (R^2^ = 0.61, r = 0.78, *p* ˂ 0.0001) (Figure 5G–I).

The different propagation methods had a significant effect on root parameters. T0 (control, whole bulb without cuts) and T1 (bulb quarters) presented the highest length, diameter, and root number. Respective values for T0 and T1 were as follows: the root diameter was 1.2 ± 0.07 mm and 1.1 ± 0.10 mm; root length was 100.74 ± 6.25 mm and 61.86 ± 1.78 mm (T0 and T1); and root number was 7.6 ± 1.67 mm and 7.4 ± 1.34 mm (Figure 4B–D). Andrade-Rodriguez et al. [12] obtained the highest number of roots with bulbs longitudinally cut into quarters.

These results indicate that T1 (cut-induced bulb division with longitudinal cuts into quarters) was the best domestication and propagation method for *H. cardenasianus* bulbs, as it resulted in the highest number of bulbils and largest bulbs, thereby ultimately producing more biomass.

### 2.4. Germination Tests

The collected seed material of *H. cardenasianus* was used in different experiments to determine the best germination conditions. The combined effect of different post-collection times (PT) and imbibition times (IT) was evaluated, resulting in 20 treatments (5 replicate and 10 seeds each): T_I_ (PT: 0 days-IT: 0 days),T_II_ (PT: 0 days-IT: 2 days),T_III_ (PT: 0 days-IT: 4 days), T_IV_ (PT: 0 days-IT: 6 days), T_V_ (PT: 0 days-IT: 8 days), T_VI_ (PT: 15 days-IT: 0 days), T_VII_ (PT: 15 days-IT: 2 days), T_VIII_ (PT: 15 days-IT: 4 days), T_IX_ (PT: 15 days-IT: 6 days), T_X_ (PT: 15 days-IT: 8 days), T_XI_ (PT: 30 days-IT: 0 days), T_XII_ (PT: 30 days-IT: 2 days), T_XIII_ (PT: 30 days-IT: 4 days), T_XIV_ (PT: 30 days-IT: 6 days), T_XV_ (PT: 30 days-IT: 8 days), T_XVI_ (PT: 45 days-IT: 0 days), T_XVII_ (PT: 45 days-IT: 2 days), T_XVIII_ (PT: 45 days-IT: 4 days), T_XIX_ (PT: 45 days-IT: 6 days), and T_XX_ (PT: 45 days-IT: 8 days). The different germination parameters were evaluated for 20 days: germination percentage, germination rate, germination period, fifty percent of seed germination time (T50), and germination energy.

The coefficients of variation were less than 10%, determination coefficients were greater than 0.90, while *p*-values of the model were less than 0.05, which indicated little variation in observations and a good fit of the statistical model by two ways ANOVA. A significant interaction (*p* < 0.05) was observed between the two factors. Therefore, the effect of the factors (PT × IT) was jointly analysed.

The obtained data demonstrated that the germination method can significantly affect all the evaluated parameters (*p* < 0.05).

In this study, all the treatments (T_I_–T_XX_) had a significant influence on the germination percentage. The highest was achieved by T_II_ (PT: 0-day and IT: 2-day) (100 ± 0.00%). The germination percentages decreased significantly as post-collection time increased (Table 2). Similarly, Santa Cruz et al. [22] reported germination percentage values of 100% for *Zephyranthes mesochloa*. However, for a species from the same genus, *Habranthus gracifolius*, the treatment without imbibition and under similar thermal-light conditions led to lower germination values (80% < %G < 90%) [14]. Herranz et al. [15] reported values higher than 90% for *Hippeastrum × johnsonii*.

The germination speed was significantly affected in the analysed conditions. TII (PT: 0-day and IT: 2-day) showed the highest germination speed with a value of 5.20 ± 0.27 seeds per day (Table 2). The germination period also differed significantly among the treatments, the shortest being 5.2 ± 0.84 days for TII (PT: 0-day and IT: 2-day) (Table 2). Similar results were reported for *H. gracilifolius*, with 6 days as the average period [14]. Significant differences were also detected regarding the fifty percent of seed germination time, T50, the lowest values being 2.00 ± 0.50 days for TII (PT: 0-day and IT: 2-day) (Table 2).

The treatments also had a significant influence on germination energy, the highest values of 68 ± 5.95 and 70 ± 5.00 days being obtained for T_II_ (PT: 0-day and IT: 2-day) and T_III_ (PT: 0-day and IT: 4-day), respectively (Table 2). In contrast, an average of 76% was reported for EG values *in H. gracifolius* [14]. Amaryllidaceae seeds, including those of *H. Cardenasianus*, have a very short longevity [5]. All the analyses of seed viability (indirectly measured by the germination parameters of this study) indicate that it decreased significantly as post-collection time increased. On the other hand, seed viability increased significantly after 2 days of imbibition, which could be explained by the removal of inhibitors from the seed by imbibition [15]. However, the seed viability significantly decreased after 4 days of imbibition due to contamination by fungi and bacteria.

The results indicate that T_II_ (post-collection time: 0-day and imbibition time: 2-day) provides the best germination conditions for *H. cardenasianus* seeds, as it produced the high germination percentage, the fastest germination speed, the shortest germination period, and the highest germination energy.

## 3. Material and Methods

### 3.1. Plant Material

Wild plants of *Habranthus cardenasianus* Traub & I.S. Nelson (seeds and bulbs) were collected at Departamento Punilla, Córdoba, Argentina during the flowering and fruiting seasons, between October 2017 and March 2018, considering a previous report on this species [5]. Plant samples were identified by MSc. German Roitman, Facultad de Agronomía, CABA (Ciudad Autónoma de Buenos Aires), Argentina. A voucher specimen was deposited at the Instituto de Biotecnología, Universidad Nacional de San Juan, San Juan, Argentina, with the code IBT-UNSJ-Arg.16.

Several bulbs were separated for chemical analysis. Before starting the propagation experiments, the collected bulbs were transferred to plastic cylindrical containers (white pots 8.5 cm in diameter and 30 cm high) with 1:1:1:1 substrate (perlite:sand:peat:organic soil) [16]. Then bulbs in cylindrical containers were kept under greenhouse conditions for 3 months and watered with Carbendazim ^®^ InsuAgro, Fragaria S.R.L., Villa Cañas, Santa Fe, Argentina) (5 g L^−1^) twice a week.

### 3.2. Alkaloid Extraction and Isolation

Dried bulbs (180 g) were extracted with MeOH (3 × 500 mL) under reflux for 1 h each. The solvent was evaporated under reduced pressure to give the methanolic crude extract (MCE). The MCE was dissolved in H_2_SO_4_ (2% *v*/*v*) and neutral material was removed with Et_2_O (250 mL). The aqueous solution was basified with 25% NH_4_OH up to pH 10–12 and the alkaloids were extracted with CHCl_3_ (3 times with 500 mL) to obtain 1.43 g of the basic chloroform extract (BCE). Before starting the isolation process, 5 mg of the BCE was dissolved in MeOH for GC/MS analysis.

The BCE was roughly separated by SiO_2_ (170 g) flash CC using an n-hexane/EtOAc/CH_2_Cl_2_/MeOH gradient to give six fractions (A−F): fractions 50 mg A (CH_2_Cl_2_), 100 mg B (CH_2_Cl_2_/EtOAc, 7:3), 114 mg C (EtOAc), 413 mg D (EtOAc/MeOH, 9:1), 244 mg E (EtOAc/MeOH, 1:1), and 50 mg F (MeOH). Column chromatography on Sephadex LH-20 (30 cm length, 2.5 cm i.d.) of fraction C in MeOH gave three subfractions (12.5 mg C1, 89 mg C2, and 6.6 mg C3). Crystallization of subfraction C2 afforded tazettine (**9**) (89 mg). Likewise, fraction D was permeated through a Sephadex LH-20 column using MeOH as the eluent to give four subfractions (64.9 mg D1, 21.5 mg D2, 191.7 mg D3, and 78.5 mg D4). The subfractions D3 and D4 were separately purified by preparative HPLC with ACN:H_2_O (3:7) as the eluent to obtain the mixture of epimers haemanthidine/6-epi-haemanthidine (10) (8 mg) and haemanthamine (8) (12 mg). For analytical TLC, CH_3_Cl/MeOH, 9:1, in NH_3_ atmosphere was used as eluent and the alkaloids bands were detected by UV light, iodine vapours resublimed, and staining with Dragendorff’s reagent.

### 3.3. Microplate Assay for AChE and BuChE Inhibitory Activities

Cholinesterase inhibitory activity assays were performed according to Ellman et al. [43] with some modifications [15]. Fifty microliters of 0.25 U/mL AChE or BuChE (Sigma-Aldrich, St. Louis, MO, USA) in phosphate buffer (8 mM K_2_HPO_4_, 2.3 mM NaH_2_PO_4_, 0.15 M NaCl, pH 7.6) and 50 μL of the sample dissolved in the same buffer were added to the wells. The plates were incubated for 30 min at room temperature before addition of 100 μL of the substrate solution (0.1 M Na_2_HPO_4_, 0.5 M 5,5′-Dithiobis(2-nitrobenzoic acid) (DTNB, Sigma-Aldrich), and 0.6 mM acetylthiocholine iodide (ATCI, Sigma-Aldrich) or butyrylthiocholine iodide (BTCI, Sigma-Aldrich) in Millipore water, pH 7.5). The absorbance at 405 nm was determined using a Thermo Scientific Multiskan FC microplate spectrophotometer (Waltham, MA, 02451, USA) after 5 min. The enzyme-inhibitory activity was calculated as a percentage compared to an assay using a buffer without any inhibitor. The enzyme-inhibitory data were analyzed using the software package Prism (Graph Pad Inc., San Diego, CA, USA). The alkaloid concentrations used to calculate the IC_50_ values were 15, 30, 60, 120, 160, and 200 μM in both AChE and BuChE assays, while for the alkaloid extract, they were 50, 100, 150, 200, 250, and 300 μg/mL, respectively. The IC_50_ values are the means ± SD of three individual determinations, each performed in triplicate.

### 3.4. Chemical Analysis

The GC-MS spectra were obtained on an Agilent 6890N GC 5975 inert MSD instrument, operating in EI mode at 70 eV (Agilent Technologies, Santa Clara, CA, USA) using a DB5-MS column (30 m × 0.25 mm × 0.25 μm). The temperature program was as follows: 100−180 °C at 15 °C min^−1^, 1 min hold at 180 °C, 180−300 °C at 5 °C min^−1^, and 10 min hold at 300 °C. The injector temperature was 280 °C. The flow rate of the He carrier gas was 0.8 mL min^−1^, and the split ratio was 1:20 (with more diluted samples, a split ratio of 1:5 was applied). A hydrocarbon mixture (C9−C36 Restek, cat no. 31614) was used for the retention index calibration. The proportion of each compound in the alkaloid fractions was expressed as a percentage of the total ion current (TIC) (Table 1). These data do not express quantification, although they can be used to compare the relative abundances of each component. The results obtained were analysed using AMDIS 2.64 software and the NIST database. Compounds were identified by comparing their mass spectral patterns and retention indices with the data recorded in the literature.

The HPLC separations were performed using HPLC-grade solvents, a Thermo Separations Spectra Series P100 pump (Middletown, VA, USA), a Thermo Separations Refractomonitor IV RI detector, a Thermo Separations SpectraSeries UV 100 UV detector, and a YMC RP-18 (5 μm, 20 mm × 250 mm) column. Sephadex LH-20 was obtained from Pharmacia Inc. (Uppsala, Sweden), and TLC was carried out on Merck silica gel 60 F_254_ plates. TLC plates were sprayed with Dragendorff reagent for alkaloid detection. Flash column chromatography (CC) was performed using Merck silica gel 60A (6−35 μm) (Darmstadt, Germany).

^1^H and ^13^C NMR data were measured in Bruker Avance II (500 MHz) and AC-200 (200 MHz) spectrometers (Bruker, Rheinstetten, Germany), in CD_3_COCD_3_. Proton chemical shifts were referenced to the residual signal of CD_3_COCD_3_ at δ 2.05, and ^13^C NMR were referenced to the central peak of CD_3_COCD_3_ at 206.26 ppm. Homonuclear ^1^H connectivities were determined by COSY experiments. The edited reverse-detected single quantum heteronuclear correlation (DEPT-HSQC) experiment allowed the determination of carbon multiplicities, as well as one-bond proton−carbon connectivities, and the heteronuclear multiple bond correlation (HMBC) experiment allowed the determination of long-range proton−carbon connectivities. The relative configuration was determined by gradient-enhanced NOESY experiments. All 2D NMR experiments were performed using standard pulse sequences (see Supplementary Materials).

### 3.5. Propagation Methods from H. cardenasianus

Different propagation methods of *H. cardenasianus* were evaluated in order to analyse biomass generation [16,20,21,42]. A completely randomized design resulted in a test of five treatments and a control, with five replicates each. A replicate was a pot with five bulbs. Vegetative propagation treatments were as follows: T0 (control, whole bulb without cuts); cut-induced bulbs: T1 (bulb quarters), T2 (twin scales), T3 (bulb basal disc); T4 (micropropagated bulbs), and T5 sexual propagation (seeds).

#### 3.5.1. Cut-Induced Bulb Division

To study cut-induced bulb division, four treatments were applied T0, T1, T2, T3. *H. cardenasianus* bulbs (30–35 mm) were prepared by removing soil, roots and external cataphylls, then disinfected with sodium hypochlorite (2.5% NaClO) for 15 min and washed with sterile water for two minutes. T1: bulbs were divided in four equal parts. T2: bulbs were divided in four equal parts and the fractions were separated into external, middle and internal twin scales. T3 consisted of a bulb cross-section with 1 cm of cataphylls above the basal disk.

The fractions obtained in each treatment and the controls were air-exposed for five days for the healing of cuts. They were then placed in plastic cylindrical containers (white pots 8.5 cm in diameter and 30 cm high) with 1:1:1:1 substrate [16]. The substrate consisted in 1 part of organic soil (loamy texture; pH: 6.7; electrical conductivity: 0.3 dS m^−1^; organic matter: 2.3%; phosphorus as phosphate: 23 ppm; nitrogen as nitrates: 42 ppm), 1 part of peat (humidity: 30–35%, ash: 5–10%, organic matter: 90–95%, carbon to nitrogen ratio C N^−1^: 38, pH: from 3.4 to 4, electrical conductivity: 0.3 dS cm^−1^), 1 part of perlite (chemically inert, pH: neutral) and 1 part of fine sand. The fractions in pots were watered with commercial fungicide Carbendazim^®^ (2 g L^−1^, active ingredient: methylbenzimidazol-2-yl carbamate). The treated plants were kept in greenhouse conditions (temperature: 15–30 °C, humidity: 60–70%).The fungicide application was repeated during the first two weeks (once a week). The plants were then watered twice a week during the seven months of experimentation.

#### 3.5.2. Micropropagation of Bulbs

To obtain micropropagated bulbs (T4), *H. cardenasianus* bulbs (30–35 mm) were exposed for five days to the air to reduce fungal contamination and then prepared by removing soil, roots and external cataphylls. The bulbs were placed in a fungicide and antibiotic solution (Carbendazim^®^ 2 g L^−1^ plus Amoxicillin 500 ppm) for 6 h and then immersed in 8% iodinated solution for 20 min before being disinfected with ethanol (70% *v*/*v*, 2 min). Finally, they were disinfected with 5% sodium hypochlorite for 10 min.

Disinfected bulbs were cut, and the twin-scales (TS) were used as primary explants [44]. TS were cultured on MS medium supplemented with 2,4-D (0.25 mg L^−1^), BAP (1 mg L^−1^), sucrose (30 g L^−1^), and Difco agar (8 g L^−1^). Then TS were kept in an incubation chamber (INGELAB, Semedic) for one week in darkness at 22 ± 1 °C to induce the formation of axillary shoots. Shoots were then re-cultured on MS medium supplemented with 2,4-D (1 mg L^−1^), BAP (4 mg L^−1^), sucrose (30 g L^−1^), and Difco agar (8 g L^−1^) for six weeks under a long-day photoperiod: 16 h of light (100 μmol m^−2^ s^−1^) at 25 ± 1 °C, and 8 h of darkness at 22 ± 1 °C [13,20,39]. Each 30 days, to avoid contamination and promote multiple shoots, bulb fractions were separated and recultured. This process was repeated for seven months.

#### 3.5.3. Seed Propagation

Good-condition seeds (color, texture, appearance) according to ISTA [45] were selected. The combined effect of 1st factor: post-collection time (PT) and 2nd factor: imbibition time (IT) were evaluated in order to determine the optimal seed germination conditions. The 1st factor had 4 levels: 0 days, 15 days, 30 days and 45 days. The 2nd factor had 5 levels: 0 days, 2 days, 4 days, 6 days, and 8 days. In this way, a completely randomized design resulted in a test of 20 treatments (4 × 5), with five replicates each. A replicate was a Petri dish with 100 seeds. The treatments are as follows: T_I_ (PT: 0 days-IT: 0 days),T_II_ (PT: 0 days-IT: 2 days),T_III_ (PT: 0 days-IT: 4 days), T_IV_ (PT: 0 days-IT: 6 days), T_V_ (PT: 0 days-IT: 8 days), T_VI_ (PT: 15 days-IT: 0 days), T_VII_ (PT: 15 days-IT: 2 days), T_VIII_ (PT: 15 days-IT: 4 days), T_IX_ (PT: 15 days-IT: 6 days), T_X_ (PT: 15 days-IT: 8 days), T_XI_ (PT: 30 days-IT: 0 days), T_XII_ (PT: 30 days-IT: 2 days), T_XIII_ (PT: 30 days-IT: 4 days), T_XIV_ (PT: 30 days-IT: 6 days), T_XV_ (PT: 30 days-IT: 8 days), T_XVI_ (PT: 45 days-IT: 0 days), T_XVII_ (PT: 45 days-IT: 2 days), T_XVIII_ (PT: 45 days-IT: 4 days), T_XIX_ (PT: 45 days-IT: 6 days), and T_XX_ (PT: 45 days-IT: 8 days). The seeds were soaked in sterile distilled water (24 h, 25 °C and dark), disinfected in ethanol (70% *v*/*v*, 5 min), and then placed in glass Petri dishes (100 × 10 mm^2^) with moistened paper (FILTER-LAB^®^ seed germination paper), using sterile distilled water. Petri dishes were kept in an incubation chamber (INGELAB, Buenos Aires, Argentina) for 30 days. An alternating temperature regime of 25 and 15 °C, light/dark cycle of 16/8 h (fluorescent tubes Philips TLD 36W/84 of white light, giving 100 μmol m^−2^ s^−1^) and 95% humidity was used as in Santa Cruz et al. [26].

In treatment 5 (T5), once the seedlings emerged, they were transferred to plastic cylindrical containers (white pots 8.5 cm in diameter and 30 cm high) with 1:1:1:1 substrate (perlite:sand:peat:organic soil) [16]. The cylindrical containers were kept in an incubation chamber for 30 days, under the same conditions as in the germination stage. After the incubation period, plants were rusticated under greenhouse conditions. They were watered with commercial fungicide Carbendazim^®^ (2 g L^−1^) twice a week during the seven months of experimentation.

### 3.6. Evaluated Parameters

Seven months after the start of the propagation test, different bulb parameters were evaluated [16,20,21,42]: survival percentage, multiplication rate, biomass increase, equatorial diameter, bulb length, root diameter, root length, root number. The following formulae were used for the quantification of the propagation parameters (1–3):

%S = Ns/Nt × 100(1)

where %S is survival; Ns is the number of surviving bulbs; and Nt is the number of cultured bulbs.

MR = Nb/Nt × 100(2)

where MR is the multiplication rate; Nb is the number of bulbils; and Nt is the number of cultured bulbs.

BI = Pf − Pi(3)

where BI is the biomass increase; Pf is the bulb dry weight at the end of the test; and Pi is the initial bulb dry weight.

Regarding the germination test, different germination parameters were evaluated for 30 days [19,26,45,46]: germination percentage, germination rate, fifty percent of seed germination time (T50), germination energy, and germination period. The following formulas were used for germination parameter quantification (1–3):

%G = S/St × 100(4)

where %G is the germination percentege; S is the germinated seeds; and St is total tested seeds.

GS = Σ(Ss/D)/Ni(5)

where GS is the germination speed; Ss is the germinated seeds for each count; D are the days from the previous count; and Ni is the count number.

The germination energy (EG%) was estimated indirectly by calculating the accumulated daily germination at the moment when most of the treatments showed the maximum daily germination speed, called the energy period (day 7 after our study started).

GP = GF − GS (6)

where GP is the germination period; GF is the day the germination finishes; and GS Ni is the day the germination starts.

### 3.7. Analysis of Data

Assumptions of normal data distribution and variance homoscedasticity were verified. Parameters evaluated by counts and percentages were transformed with the √x function, prior to statistical analysis. Propagation method data were analysed by analysis of variance (ANOVA). The effect of treatments was considered. In the case of a significant F-value, the means were compared with Tukey’s test at the significance level of *p* < 0.05. Standard deviation data were included. A linear correlation analysis between root parameters (length, diameter and root number) and biomass parameters (equatorial diameter, length and bulb dry weight) was applied. The coefficient of determination (R^2^), the Pearson’s correlation coefficient (r) and the *p* values are reported. Germination method data were analysed by two ways ANOVA to evaluate simultaneously the effect of two grouping variables (PT and IT) on a response variable. In the case of a significant F-value, the means were compared with Tukey’s test at the significance level of *p* < 0.05. Standard deviation data were included. InfoStat software 2017 was used [47].

## 4. Conclusions

The interest in Amaryllidaceae alkaloids has increased in recent years because of their wide range of bioactivities. Despite their promising pharmacological potential, the clinical development of Amaryllidaceae alkaloids is hindered by the limited quantities available in natural sources. The phytochemical analyses of *H. cardenasianus* bulbs showed that they are a source of antitumoral alkaloids, especially tazettine (9) (pretazettine). It can be concluded that the lack of inhibitory activities of the alkaloid extract towards cholinesterases (AChE and BChE) might be due to the absence of galathamine-type alkaloids in this species. Regarding the sexual propagation, the best conditions for *H. cardenasianus* seeds were 0-day post-collection and two days of imbibition, whereas T1 was the best vegetative propagation method, providing the highest number of and biggest bulbs, therefore ultimately producing more biomass. It can be affirmed that the cut-induced bulb division with longitudinal cuts into quarters (T1) is a viable and sustainable alternative for the propagation and domestication of *H. cardenasianus* as a source of promising bioactive alkaloids.

## Figures and Tables

**Table 1:** GC-MS data for *H. cardenasianus* alkaloid extract.

Alkaloid	[M^+^]	RI ^1^	TIC ^2^	MS
**Galanthindole-type**				
Galanthindole (**4**)	281(100)	22.8	0.86	264(13), 263(16), 262(18), 252(15), 248(8), 191(11), 132(11), 107(8)
**Haemanthamine-type**				
Haemanthamine (**8**)	301(11)	24.9	7.92	273(19), 272(100), 242(13), 240(14), 214(14), 212(13), 211(15), 186(14), 181(25), 128(13)
Haemanthidine/ 6-*epi*-haemanthidine (**10**) ^3^	317(94)	26.2	6.68	284(80), 233(77), 211(53), 201(100), 199(77), 181(71), 173(64), 115(79), 45(53)
**Homolycorine-type**				
Unknown (**3**)	331(5)	22.4	0.82	330(2), 329(6), 221(4), 110(8), 109(100), 108(13), 94(2), 82(2), 81(1), 42(2)
Unknown (**6**)	331(<1)	23.2	4.29	221(7), 191(1), 163(1), 110(8), 109(100), 108(13), 94(2), 82(2), 81(1), 42(1)
**Ismine-type**				
Ismine (**1**)	257(30)	19.3	0.701	239(20), 238(100), 211(6), 196(9), 180(8), 168(7), 167(6), 152(5), 139(6)
**Lycorine-type**				
Anhydrolycorine (**5**)	251(41)	23	0.97	250(100), 203(6), 192(10), 130 (15), 129(5), 118(4), 57(6), 41(5)
11,12-dehydro anhydrolycorine (**7**)	249(61)	24.5	2.70	248(100), 191(10), 190(24), 189(6), 164(3), 163(7), 123(6), 95(14)
Lycorine (**11**)	287(24)	26.7	2.55	286(19), 268(24), 250(15), 227(79), 226(100), 211(7), 147(15)
**Narciclasine-type**				
5,6-Dihydrobicolorine (**2**)	239(44)	20.2	1.90	238(100), 181(3), 180(10), 166(3), 152(5), 139(6), 118(6), 90(7)
**Pretazettine-type**				
Tazettine (**9**)	331(26)	25.3	57.77	298(19), 248(15), 247(100), 201(16), 199(14), 181(13), 115(15), 71(15), 70(17)

^1^ Kovats retention index; ^2^ Total ion current; ^3^ cannot be distinguished by GC-MS.

**Table 2:** Effects of the imbibition time (ImbT) and post-collection time on germination parameters of germination percentage, germination speed, germination period, T_50_ and germination energy.

	Post-Collection Time								
P	ImbT D	Days							
		0		15		30		45	
%G	0	T_I_	92.00 ± 4.47 ^I^	T_VI_	76.00 ± 5.48 ^H^	T_XI_	36.00 ± 5.48 ^DE^	T_XVI_	6.00 ± 0.48 ^AB^
	2	T_II_	100.00 ± 0.00 ^J^	T_VII_	82.00 ± 4.47 ^HI^	T_XII_	44.00 ± 5.48 ^EF^	T_XVII_	8.00 ± 0.75 ^B^
	4	T_III_	94.00 ± 5.48 ^I^	T_VIII_	78.00 ± 4.47 ^H^	T_XIII_	40.00 ± 7.07 ^E^	T_XVIII_	4.00 ± 0.48 ^AB^
	6	T_IV_	84.00 ± 5.48 ^HI^	T_IX_	64.00 ± 5.48 ^G^	T_XIV_	26.00 ± 5.48 ^D^	T_XIX_	2.00 ± 0.25 ^A^
	8	T_V_	58.00 ± 8.37 ^F^	T_X_	44.00 ± 5.48 ^EF^	T_XV_	18.00 ± 4.37 ^C^	T_XX_	2.00 ± 0.25 ^A^
GS	0	T_I_	4.70 ± 0.27 ^G^^H^	T_VI_	3.80 ± 0.27 ^F^	T_XI_	1.80 ± 0.27 ^D^	T_XVI_	0.40 ± 0.12 ^AB^
	2	T_II_	5.20 ± 0.27 ^H^	T_VII_	4.20 ± 0.27 ^FG^	T_XII_	2.20 ± 0.27 ^DE^	T_XVII_	0.40 ± 0.12 ^AB^
	4	T_III_	4.80 ± 0.45 ^GH^	T_VIII_	3.90 ± 0.22 ^F^	T_XIII_	2.10 ± 0.22 ^DE^	T_XVIII_	0.20 ± 0.04 ^A^
	6	T_IV_	4.40 ± 0.22 ^G^	T_IX_	3.40 ± 0.42 ^EF^	T_XIV_	1.40 ± 0.42 ^C^	T_XIX_	0.10 ± 0.03 ^A^
	8	T_V_	3.10 ± 0.65 ^E^	T_X_	2.30 ± 0.27 ^DE^	T_XV_	0.9 ± 0.42 ^B^	T_XX_	0.10 ± 0.02 ^A^
GP	0	T_I_	8.00 ± 0.71 ^AB^	T_VI_	11.60 ± 1.14 ^BC^	T_XI_	13.60 ± 0.55 ^CD^	T_XVI_	17.40 ± 2.61 ^EF^
	2	T_II_	5.20 ± 0.84 ^A^	T_VII_	10.60 ± 0.55 ^B^	T_XII_	16.00 ± 2.12 ^DE^	T_XVII_	16.60 ± 3.29 ^E^
	4	T_III_	8.40 ± 0.55 ^AB^	T_VIII_	12.80 ± 0.84 ^C^	T_XIII_	14.80 ± 1.48 ^D^	T_XVIII_	18.00 ± 2.12 ^F^
	6	T_IV_	12.40 ± 1.14 ^C^	T_IX_	14.40 ± 0.55 ^D^	T_XIV_	16.00 ± 1.00 ^DE^	T_XIX_	19.20 ± 1.79 ^GH^
	8	T_V_	15.40 ± 1.34 ^DE^	T_X_	15.80 ± 0.84 ^DE^	T_XV_	15.80 ± 2.28 ^DE^	T_XX_	19.60 ± 0.89 ^H^
T_50_	0	T_I_	4.40 ± 1.14 ^AB^	T_VI_	6.80 ± 1.05 ^C^	T_XI_	7.60 ± 2.02 ^CD^	T_XVI_	8.60 ± 2.61 ^DE^
	2	T_II_	2.00 ± 0.50 ^A^	T_VII_	5.40 ± 1.52 ^B^	T_XII_	8.40 ± 2.28 ^DC^	T_XVII_	8.60 ± 1.82 ^DE^
	4	T_III_	5.00 ± 0.50 ^B^	T_VIII_	6.20 ± 1.92 ^BC^	T_XIII_	7.00 ± 1.92 ^C^	T_XVIII_	9.80 ± 1.79 ^E^
	6	T_IV_	6.80 ± 1.30 ^BC^	T_IX_	7.80 ± 1.30 ^D^	T_XIV_	7.20 ± 0.84 ^C^	T_XIX_	10.20 ± 1.79 ^EF^
	8	T_V_	8.40 ± 2.16 ^C^	T_X_	8.00 ± 1.55 ^D^	T_XV_	8.00 ± 1.58 ^D^	T_XX_	10.60 ± 1.34 ^F^
GE (%)	0	T_I_	62.00 ± 5.95 ^GH^	T_VI_	58.00 ± 4.83 ^G^	T_XI_	42.00 ± 4.47 ^E^	T_XVI_	6.00 ± 1.48 ^AB^
	2	T_II_	68.00 ± 5.95 ^H^	T_VII_	54.00 ± 3.42 ^F^	T_XII_	42.00 ± 4.37 ^E^	T_XVII_	8.00 ± 1.48 ^B^
	4	T_III_	70.00 ± 5.00 ^H^	T_VIII_	56.00 ± 8.17 ^FG^	T_XIII_	44.00 ± 4.48 ^EF^	T_XVIII_	4.00 ± 0.47 ^A^
	6	T_IV_	54.00 ± 5.17 ^F^	T_IX_	54.00 ± 5.17 ^F^	T_XIV_	26.00 ± 5.40 ^D^	T_XIX_	2.00 ± 0.47 ^A^
	8	T_V_	26.00 ± 2.95 ^D^	T_X_	26.00 ± 2.40 ^D^	T_XV_	14.00 ± 5.48 ^C^	T_XX_	2.00 ± 0.47 ^A^

**ImbT:** imbibition time **P:** Parameters; **D:** Days; **%G**: Germination percentage; **GS:** Germination Speed (seeds/day); **GP:** Germination Period (days); **T_50_** (days); **GE:** Germination Energy (%). Means and standard deviation are reported. Data were analyzed by ANOVA and different letters indicate significant differences by the Tukey test (*p* < 0.05).

## Data Availability

The data presented in this study are openly available in at DOI reference number

## Supplementary Materials

The supplementary material contains ^1^H, ^13^C, and DEPT NMR spectra for the isolated compounds.

## Sample Availability

Samples of the compounds 8 and 9 are available from the authors.
