# Supplementary material for: Alkaloids Analysis of Habranthus cardenasianus (Amaryllidaceae), Anti-Cholinesterase Activity and Biomass Production by Propagation Strategies
Source: Molecules. 2021 Jan 2;26(1):192. doi: 10.3390/molecules26010192 (PMC7795992; doi:10.3390/molecules26010192)

Supplementary Material

Alkaloids Analysis of *Habranthus cardenasianus* (Amaryllidaceae), anti-cholinesterase activity and Biomass Production by Propagation Strategies

Contents:

Figure S1. ^{1}H NMR (500MHz, ClCD$_3$) spectrum of haemanthidine/6-epihaemanthidine

Figure S2. ^{13}C NMR and DEPT (125MHz, ClCD$_3$) spectra of haemanthidine/6-epihaemanthidine

Figure S3. ^{1}H NMR (500MHz, ClCD$_3$) spectrum of haemanthamine

Figure S4. ^{13}C NMR and DEPT (125MHz, ClCD$_3$) spectra of haemanthamine

Figure S5. ^{1}H NMR (500MHz, ClCD$_3$) spectrum of tazettine

Figure S6. ^{13}C NMR and DEPT (125MHz, ClCD$_3$) spectra of tazettine

Figure S1. ^{1}H NMR (500MHz, $ClCD_3$) spectrum of haemanthidine/6-epihaemanthidine

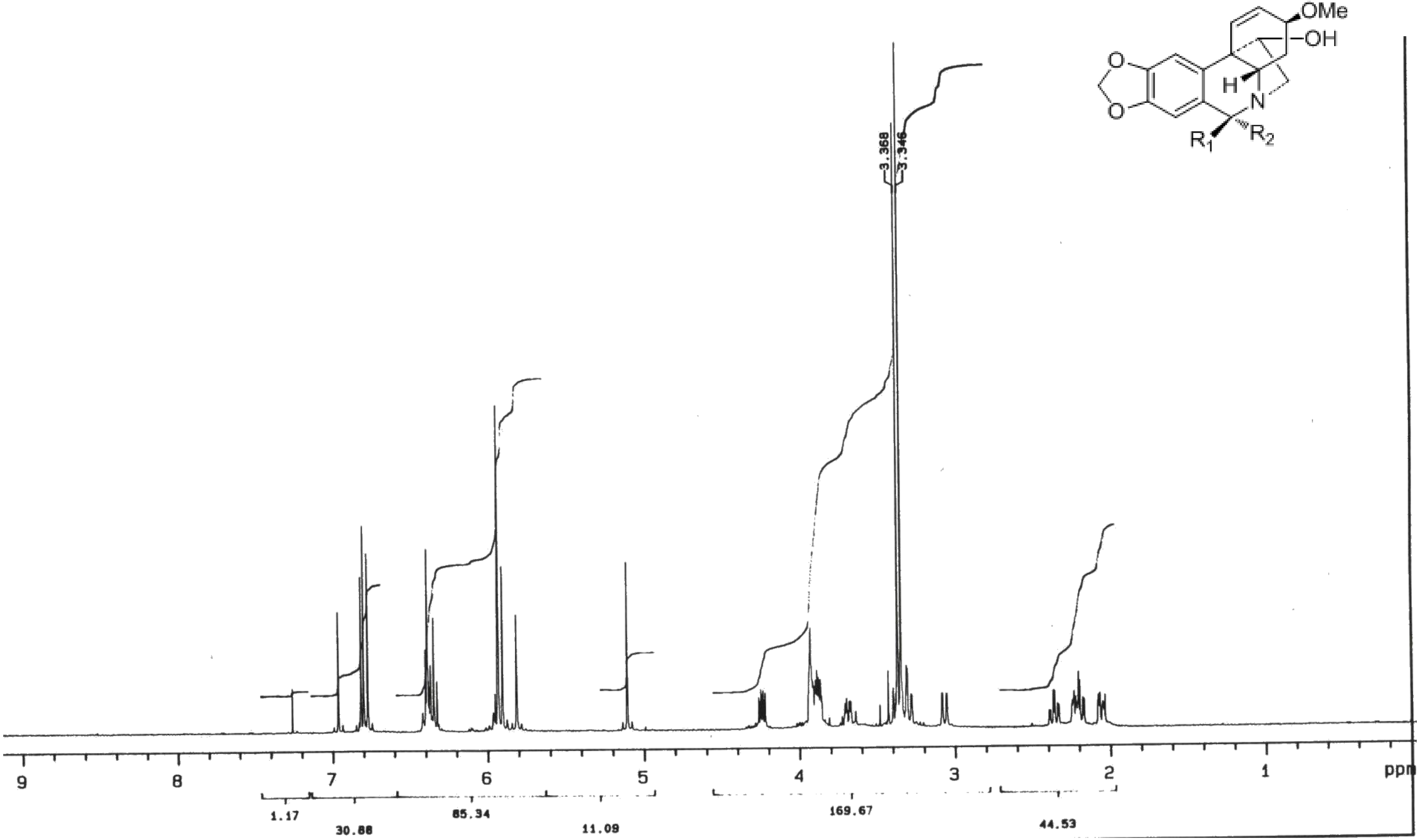

Figure S2. ^{13}C NMR and DEPT (125MHz, $ClCD_3$) spectra of haemanthidine/6-epihaemanthidine

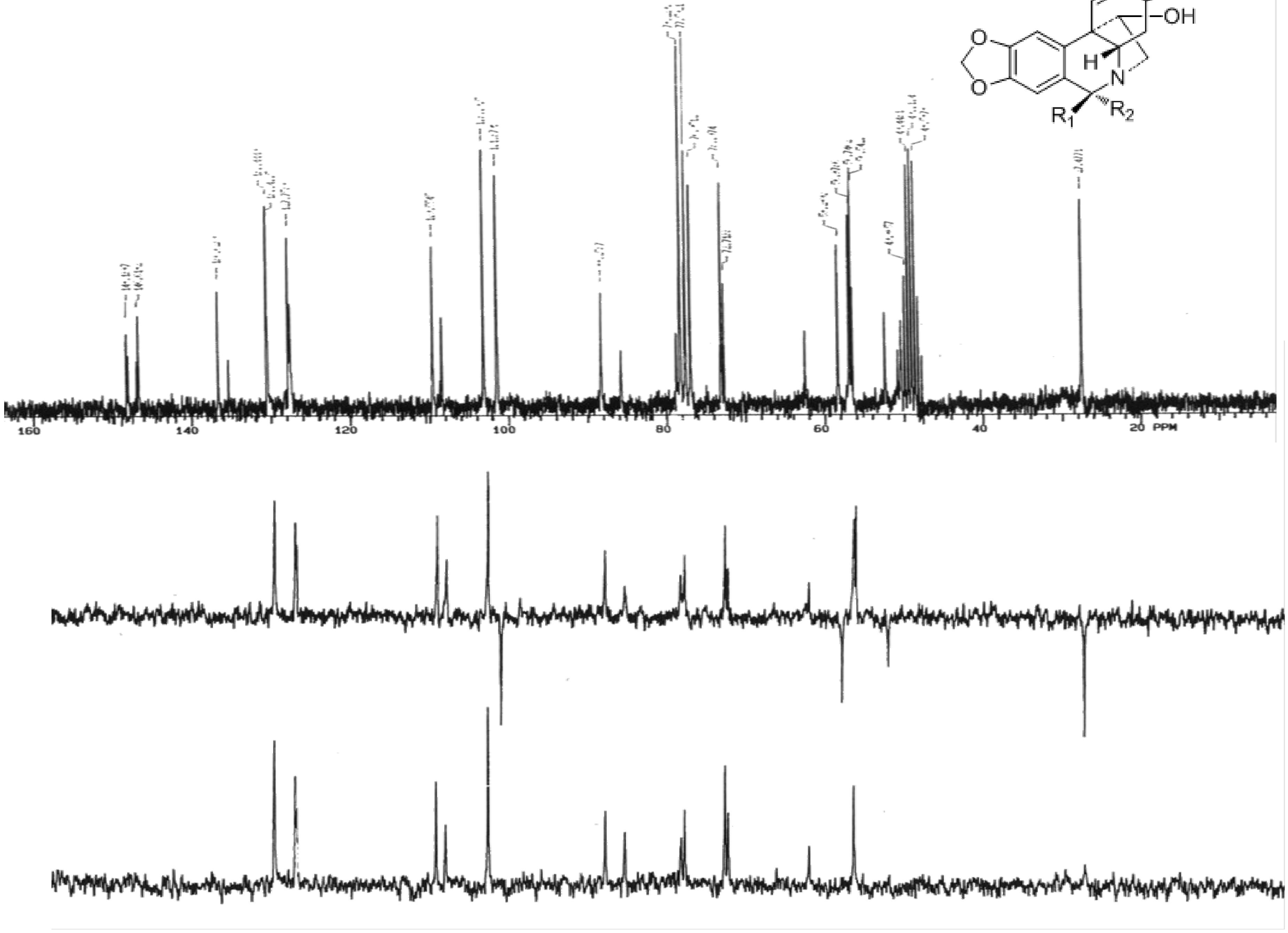

Figure S3. ^{1}H NMR (500MHz, $ClCD_3$) spectrum of haemanthamine

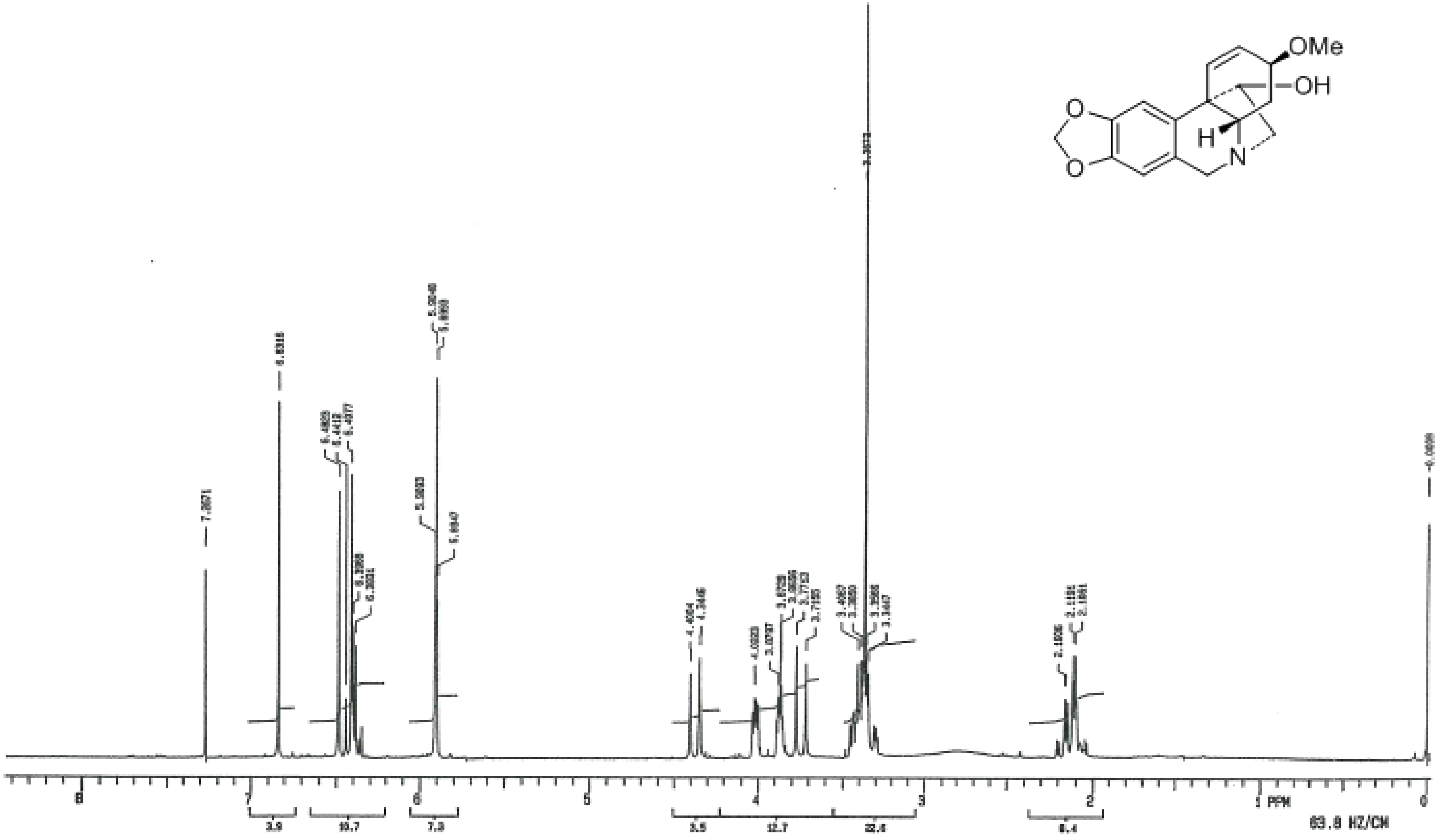

Figure S4. ^{13}C NMR and DEPT (125MHz, $ClCD_3$) spectra of haemanthamine

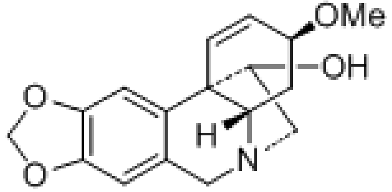

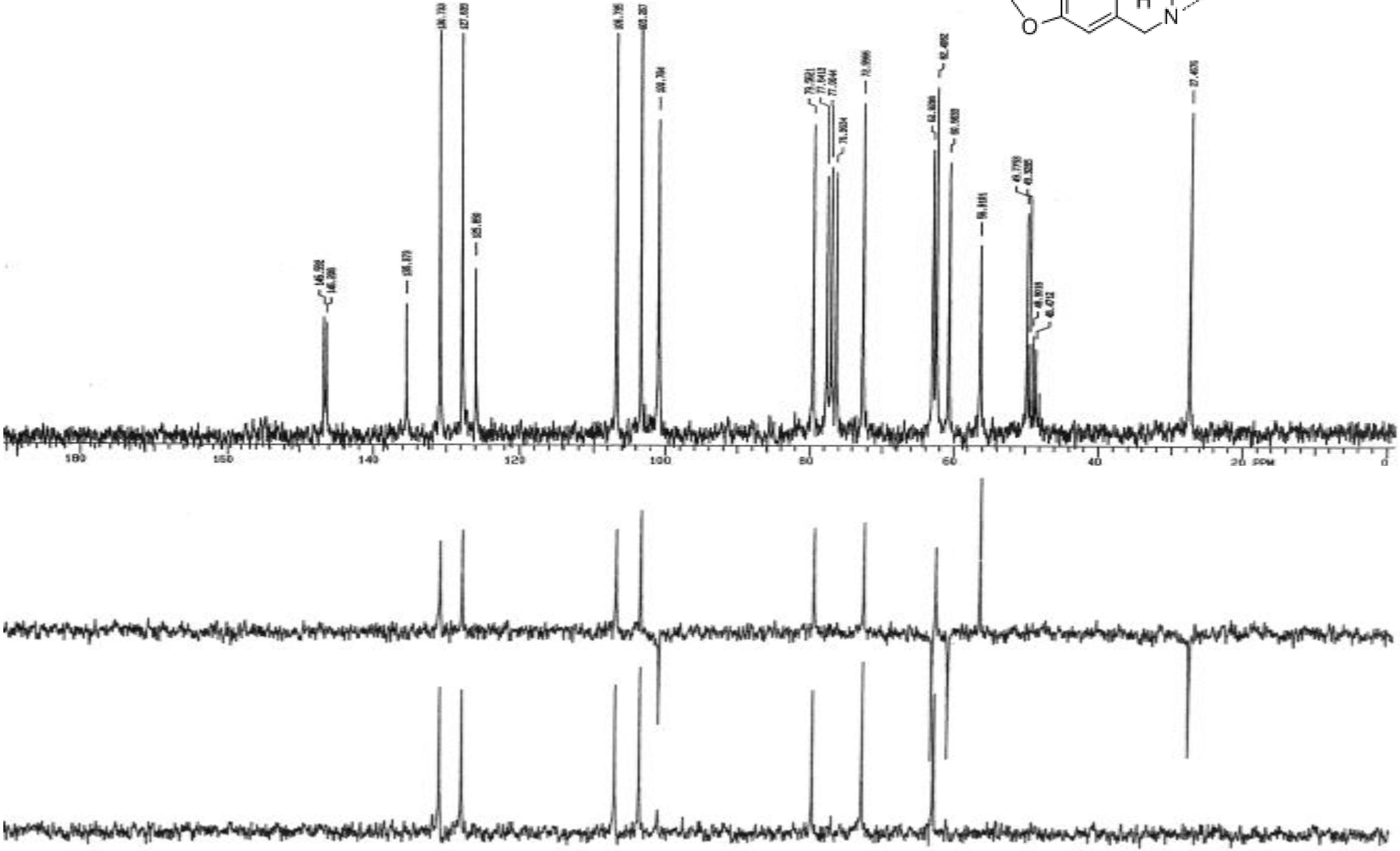

Figure S5. ^{1}H NMR (500MHz, ClCD$_3$) spectrum of tazettine

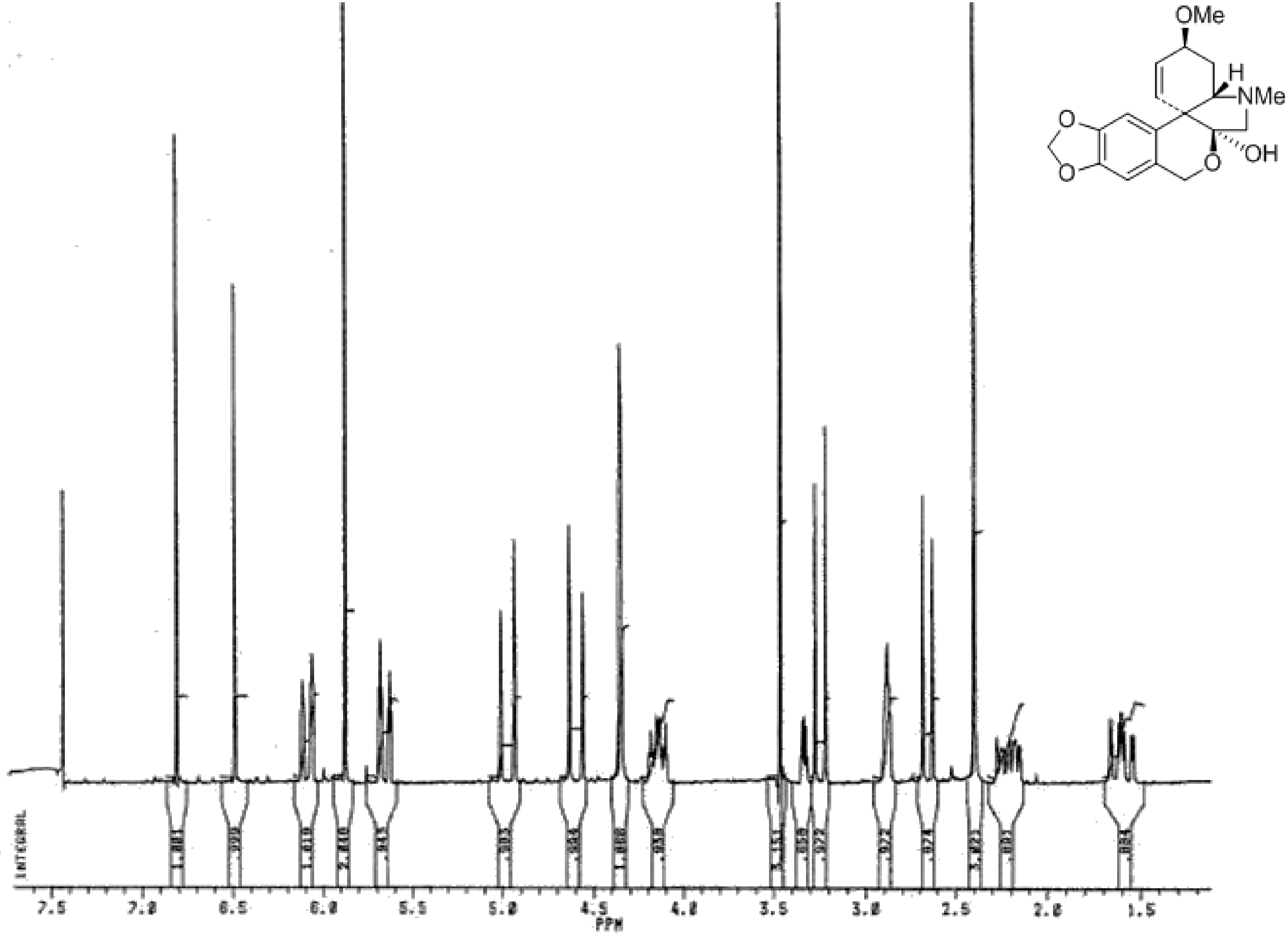

Figure S6. ^{13}C NMR and DEPT (125MHz, $ClCD_3$) spectra of tazettine

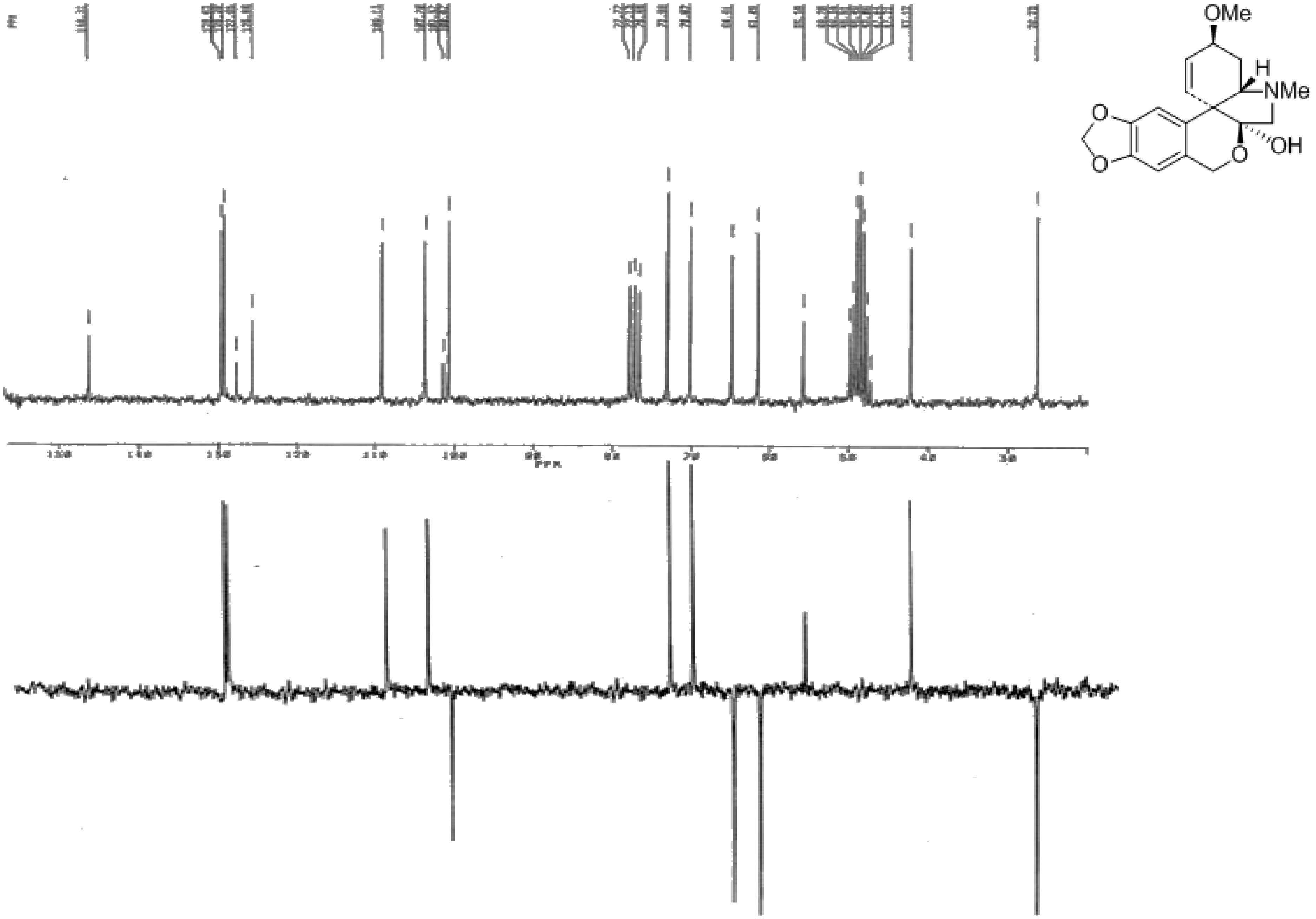

Supplement: Supplementary file 1 [file molecules-26-00192-s001.pdf]
